# The effect of high dose antibiotic impregnated cement on rate of surgical site infection after hip hemiarthroplasty for fractured neck of femur: a protocol for a double-blind quasi randomised controlled trial

**DOI:** 10.1186/1471-2474-14-356

**Published:** 2013-12-17

**Authors:** Andrew P Sprowson, Cyrus D Jensen, Sanjay Gupta, Nick Parsons, Aradhyula N Murty, Simon MG Jones, Dominic Inman, Mike R Reed

**Affiliations:** 1 Warwick Medical School, The University of Warwick, Coventry CV4 7AL, UK; 2 Northumbria NHS trust, Rake Lane, North Shields, Tyne and Wear NE29 8NH, UK

## Abstract

**Background:**

Mortality following hip hemiarthroplasty is in the range of 10-40% in the first year, with much attributed to post-operative complications. One such complication is surgical site infection (SSI), which at the start of this trial affected 4.68% of patients in the UK having this operation. Compared to SSI rates of elective hip surgery, at less than 1%, this figure is elevated. The aim of this quasi randomised controlled trial (RCT) is to determine if high dose antibiotic impregnated cement can reduce the SSI in patients at 12-months after hemiarthroplasty for intracapsular fractured neck of femur.

**Methods:**

848 patients with an intracapsular fractured neck of femur requiring a hip hemiarthroplasty are been recruited into this two-centre double-blind quasi RCT. Participants were recruited before surgery and quasi randomised to standard care or intervention group. Participants, statistician and outcome assessors were blind to treatment allocation throughout the study. The intervention consisted of high dose antibiotic impregnated cement consisting of 1 gram Clindamycin and 1 gram of Gentamicin. The primary outcome is Health Protection Agency (HPA) defined deep surgical site infection at 12 months. Secondary outcomes include HPA defined superficial surgical site infection at 30 days, 30 and 90-day mortality, length of hospital stay, critical care stay, and complications.

**Discussion:**

Large randomised controlled trials assessing the effectiveness of a surgical intervention are uncommon, particularly in the speciality of orthopaedics. The results from this trial will inform evidence-based recommendations for antibiotic impregnated cement in the management of patients with a fractured neck of femur undergoing a hip hemiarthroplasty. If high dose antibiotic impregnated cement is found to be an effective intervention, implementation into clinical practice could improve long-term outcomes for patients undergoing hip hemiarthroplasty.

**Trial registration:**

Current Controlled Trials: ISRCTN25633145

## Background

With an increasingly elderly and growing population, the number of osteoporotic fractures will steadily increase over the next few decades [1]. In the UK over the next 40 years, the number of people over 60 years will rise by around 50%, and the number of those over 90 years of age will double [2]. A neck of femur fracture (NOF) is one of the most common emergency surgical procedures, often as a result of osteoporosis, with an estimate of 120,000 occurring annually by 2015 in the National Health Service (NHS) [3]. Direct medical costs to the UK healthcare economy were £1.8 billion in 2000, with the potential to increase to £2.2 billion by 2025, with most of these costs relating directly to post-operative care [4]. With this comes an increased clinical workload of patients with NOF fractures, and subsequent complications secondary to surgery.

These patients can be broadly split into two distinct groups, based on the fracture site and subsequent treatment; (i) intertrochanteric fractures (extracapsular), treated with dynamic hip screw or cannulated screws and (ii) intracapsular NOF fractures, which if displaced are either treated with a total hip replacement (THR), bipolar prosthesis (BP) or hemiarthroplasty. Based on the 2012 National Hip Fracture Database (NHFD) figures 23% of undisplaced and 60.7% of displaced intracapsular fractured NOF were treated with cemented hemiarthroplasties [5].

Dr Foster Intelligence published a Good Hospital Guide in 2005/6, which found that there were 68,000 hip fracture patients treated that year, with mortality being 10% at 30 days, 20% at 4 months and 30% at 1 year after admission. The global literature confirms rates in the range 10-40% in the first year [6-8], with much of this attributed to post-operative complications. One such complication is SSI, which was documented on commencement of this trial at 4.08% in patients undergoing this procedure based on the 2007 HPA report [9]. Compared to complication rates of elective hip surgery at 0.91%, the figure is over four times higher. It has been suggested in the literature that the higher rate is due to increased age and other underlying co morbidities. However a recent large study looked at 169,145 fracture cases and 524,010 controls [10], and concluded that patients with a hip fracture have a pronounced excess mortality risk linked to the fracture event and postoperative complication and not to pre-existing co-morbidity. In addition, a patient’s post-operative stay in hospital for this procedure is longer than those undergoing elective hip or knee replacement and therefore the chance of getting an infection is thought to increase [11]. This therefore indicates that as healthcare providers, we should aim to target a decrease in postoperative complications.

One well established approach in orthopaedics is the use of various antibiotics. Antibiotics, such as gentamicin are commonly administered locally (in the cement) or parenterally. In a review of 15000 primary total hip replacements from the Norwegian Arthroplasty Register the lowest risk of revision was found in patients who received both systemic and local (in cement) antibiotics [12]. Use of parenteral and antibiotics in cement is standard practice in the UK for cemented hemiarthroplasty [4]. This trial aims to establish if a high dose antibiotic regime has fewer infections compared to normal dose antibiotic cement.

## Methods

The Fractured Hip Infection Trial (FHIT) is an on-going two-centre double blind quasi-randomised controlled trial conducted at Northumbria NHS Foundation Trust, one of the largest elective orthopaedic centres in the UK. The study has been approved by Newcastle and North Tyneside Research Ethics Committee [2] (07/H0901/63) and all participants provide informed, written consent. Specific permission was gained for patients with impaired mental capacity. The trial has been registered with Current Controlled Trials (ISRCTN25633145).

### Study duration

Recruitment into the trial began in November 2008, all participants are now recruited and the 12-month follow-up stage will be completed in 2013.

### Participant recruitment

Potential participants were screened and recruited by research associates or trained medical staff using Good Clinical Practice (GCP) the following morning after admission. Capacity to give consent was assessed on admission to the orthopaedic ward and at the time consent was taken. Those participants with normal mental capacity underwent standard consent having their preferences discussed and their agreement to treatment sought. Non capable participants had consent gained by their next of kin or, if they were unavailable, by a senior member of nursing staff, not involved in the study. The decision regarding capacity was made by a senior member of medical staff and confirmed by the treating orthopaedic consultant. If the participant lacked capacity and had not previously expressed their wishes, their fractured neck of femur was treated in line with national practice. Inclusion criteria include being listed for a hip hemiarthroplasty for fractured neck of femur and being willing to provide fully informed consent, or fulfil the above-defined criteria. In this pragmatic trial setting, inclusion criteria stated any patient with an intracapsular fractured neck of femur deemed suitable for a hip hemiarthroplasty and the only exclusion criterion is that patients must not be under 18 years of age. In order to explore generalisability (the patients enrolled in the study being representative of those undergoing hip hemiarthroplasty) anonymised data about age and gender was recorded for all eligible patients.

### Participant allocation

Frail patients sustain a fractured neck of femur at random times and are transferred along geographical boundaries to one of the two participating hospitals. Both units undertake surgery with >95% of these patients undergoing surgery within 48 hours (NHFD report 2012), with timely surgery being incentivised by payments of £1335 per patient [5]. This approach ensured the surgeon had little capacity to influence participant treatment allocation. Treatment group allocation was based upon the date surgery was performed. This allocation was based on a monthly hospital assignment into one of the two groups, each centre providing one treatment for the whole calendar month. The following month this process was reversed to ensure comparable groups. A more optimal conventional randomisation methodology, that is randomly allocating individual study participants to one of the two treatment groups at recruitment, was not possible for this study. It was decided by the study development team that it would not be feasible or practical to attempt to do this in the selected setting. Lack of specific local support for randomisation and concerns about the impact on the credibility and fidelity of the study interventions were identified as problematic issues if individual participant randomisation were adopted. The selected quasi randomisation approach, where treatment allocation was based on the month surgery was undertaken, although not as rigorous, was selected as the only practical option for this study. Due to the unpredictable nature of the injury and short time interval between injury and surgery, there was no reason to believe that the selected method of treatment allocation would impact on treatment effect estimates. Within this study setting it was impossible to blind the operating surgeon, as an experienced surgeon may be able to tell the difference between the cement, even though the characteristics are very similar, the handling characteristics could potentially be different and the surgeon would need to be aware. Additionally the boxes were not blinded and therefore the surgeon could see the type of cement used. The study participants, research nurses involved in recruitment and assessment, and clinical staffs involved in the care of study participants were all blind to treatment allocation throughout the study.

### Interventions

#### Hip hemiarthroplasty participants

Before the operation, all patients followed the same pathway from Accident and Emergency to the ward and the same preoperative optimisation process prior to the operation. To ensure that the trial results could be generalised to as wide a group of patients as possible, each patient had the allocated surgery according to the preferred technique of the operating surgeon. Prophylactic parenteral antibiotics prophylaxis at the start of trial was Gentamicin (4.5 mg/kg) and this was changed to Gentamicin (3 mg/kg) and Teicoplanin (400 mg) in February 2009 [13] in line with our trust prophylaxis for primary joint replacement. Antibiotics were given as a single dose, within 30 minutes of induction.

### Standard care group

This study is pragmatic in design and we did not stipulate methods of analgesia and anaesthesia, or post-operative care. A standard hemiarthroplasty implant was used in every case. Methylmethacrylate cement with low dose antibiotics (0.5 g of Gentamicin) with normal viscosity was inserted using a simple retrograde technique, with a cement restrictor. In order to maintain consistency of antibiotic release a commercial product was used, rather than the antibiotic being added by the surgeon [14].

### Intervention group

The intervention group received exactly the same regimen as the standard of care group, except they receive Methylmethacrylate cement impregnated with high dose antibiotics. This cement consists of 1 g Clindamycin and 1 g of Gentamicin. The cement is a commercial product, from the same company to maintain consistency of antibiotic release [15].

### Risks

No additional risks for study patients were expected, since all surgical procedures carried out within FHIT represent clinically established standard methods of treatment of fractured neck of femur.

### Primary outcome measure

The primary outcome is SSI infection based upon Health Protection Agency (HPA) published definitions, which originate from the Centers for Disease Control and Prevention (CDC) 1992 published definition. The HPA criteria are the nationally agreed definition used within the UK, and routinely collected by the majority of UK Trusts. Deep incisional infection is defined as a surgical site infection involving the deep tissues (i.e. fascial and muscle layers) that occurs within 30 days of surgery if no implant is in place, or within a year if an implant is in place, the infection appears to be related to the surgical procedure, and meets at least one of the criteria in Table 1.

**Table 1:** Health protection agency definition of superficial and deep surgical site infection

**Superficial incisional infection**	**Deep incisional infection**
SSI that occurs within 30 days of surgery, involves only the skin or subcutaneous tissue of the incision & meets at least one of the following criteria:	SSI involving the deep tissues (i.e. fascial & muscle layers), within 30 days of surgery (or 1 year if an implant is in place) and the infection appears to be related to the surgical procedure & meets at least one of the following criteria:
1. Purulent drainage from superficial incision	1. Purulent drainage from deep incision (not organ space)
2. Culture of organisms and pus cells present:	2. Organisms from culture and pus cells present in:
fluid / tissue from superficial incision wound swab from superficial incision	Fluid / tissue from deep incision or wound swab from deep incision
3. At least 2 symptoms of inflammation:	3. Deep incision dehisces or deliberately opened and patient has at least 1 symptom of:
Pain, tenderness, localised swelling, redness, heat and either:	
1) Incision deliberately opened to manage infection	Fever or localised pain/tenderness
OR	4. Abscess or other evidence of infection in deep incision:
	Re-operation / histopathology / radiology
2) Clinicians diagnosis of superficial SSI	5. Clinicians diagnosis of deep incisional SSI
Note: Stitch abscesses (minimal inflammation/discharge at suture point) do not classify as SSI	Note: An infection involving both superficial and deep incisional = deep incisional inflammation/discharge at suture point) do not classify as SSI

### Secondary outcome measures

Superficial incisional infection is defined by the HPA, as a surgical site infection that occurs within 30 days of surgery and involves only the skin or subcutaneous tissue of the incision, and meets at least one of the criteria in Table 1.

### Length of hospital stay

This is calculated from participant’s admission and discharge dates.

### 30 and 90-day mortality

Mortality data were obtained from the Office of National Statistics (ONS). In England, deaths must be registered within 5 days. Burials and cremations cannot be conducted without this registration documentation. These deaths are recorded by the ONS and are added to the patient’s health service record.

### Medical and surgical complications

This data is recorded from hospital records during the in-patient stay, and utilising Hospital Episode Statistics data for readmission, further surgical admission, re operation up to 12-months post-operative. If participants report the occurrence of a complication at any point within the 12- month’s post-operative, this is verified by a review of their hospital records.

### Pre operative patient factors

A number of possible potential prognostic factors are being recorded, including socio-demographic factors and medical co-morbidities.

### Specific post operative complications

An additional number of specific complications are being recorded such as Deep Vein Thrombosis (DVT) at 60 days, Pulmonary Embolism (PE) at 60 days, stroke at 30 days, Transient Ischemic Attack (TIA) at 30 days, GI Bleed at 30 days (GIB), Urinary Retention at 30 days (UR), Urinary tract infection (UTI) at 30 days, Myocardial Infarction (MI) at 30 days, Pneumonia at 30 days (RTI), and readmission rate.

Acute Renal Failure at 30 days (ARF) and C. Difficile rates will also be recorded, due to potential adverse events related to higher doses of antibiotics impregnated within the intervention cement.

### Delay to surgery

Delay to surgery was measured in both groups to ensure this was unaffected by treatment allocation.

### Sample size calculation

The primary outcome for this trial is deep SSI based on the HPA defined criteria at 12-months post-operation. Eight hundred and forty eight patients listed for hip hemiarthroplasty are being recruited into the study, and quasi randomised to the intervention arm or the standard care arm. At the initiation of the study the national rate of SSI was 4.68% for hip hemiarthroplasty, based upon Health Protection Agency data. The research team also analyzed the organisms causing infection within this setting, and the border spectrum of antibiotics covered 90% of these organisms. This sample size will provide 80% power to detect a reduction of SSI infection from 4% to 1%, at the 5% level. This significant effect size has been documented within our intervention cement in infected single stage revisions where it is able to eradicate infections in about 80% of cases. This difference represents a significant reduction, which would have an important clinical impact.

### Statistical analysis

A CONSORT diagram [16] will summarise participant flow through the study, documenting eligibility and recruitment, receipt of intervention or standard care as allocated, and collection of data [17]. Standard statistical summaries (e.g. medians and ranges or means and variances, dependent on the distribution of the outcome) and graphical plots showing correlations will be presented for the primary outcome measure and all secondary outcome measures. Baseline data will be summarised to check comparability between treatment arms, and to highlight any characteristic differences between those individuals in the study, those ineligible, and those eligible but withholding consent. Although missing data is not expected to be a major issue for this study, the nature and pattern of the missingness will be carefully considered — including in particular whether data can be treated as missing completely at random (MCAR). If judged appropriate, missing data will be imputed, and resulting imputed datasets will be analysed and reported, together with appropriate sensitivity analyses. Formal analysis, for example using logistic regression with ‘missingness’ as a response, may also be appropriate and aid interpretation.

The primary outcome, deep infections, will be compared between treatment groups using logistic regression analysis, adjusting for both participant age and gender; regression coefficients will be considered to be significant if p-values are less than 0.05 (5% significance level). Estimates of treatment effects will be presented with 95% confidence intervals. Although generally we have no reason to expect that the clustering effects will be important for this study, in reality the data will be hierarchical in nature, with patients naturally clustered into groups by operating surgeon. Therefore we propose to account for this by generalizing the conventional linear (fixed-effects) logistic regression approach to a more general mixed-effects modelling approach where patients are naturally grouped by surgeons; i.e. a random effect is included in the model to account for heterogeneity due to the operating surgeon. This analysis will be presented in addition to the conventional fixed effects model. All analyses will be conducted on an intention-to-treat (ITT) basis, and reported as such; additional per-protocol analyses will be undertaken and reported if these prove to be informative.

Any subsequent amendments to this initial SAP will be clearly stated and justified. Interim analyses will be performed only where directed by the data monitoring committee (DMC). The routine statistical analysis will mainly be carried out using R (http://www.r-project.org/) and S-PLUS (http://www.insightful.com/).

## Discussion

Surgical site infection in this vulnerable group of patients is associated with increased mortality, morbidity, and pain, which are well documented, in the international literature [18]. For patients undergoing this procedure, reduction of postoperative complications should be a targeted objective for orthopaedic surgeons [19].

There is great heterogeneity relating to patient pathways once a fractured NOF has occurred, making a suitable intervention difficult to isolate. A targeted intervention ideally needs to be easily adopted, low-intensity, independent of local hospital policy, and surgeon factors [20]. One of the great potencies of this trial is the ease with which the intervention could be applied pragmatically into any hospital in the NHS.

The number of patients who develop deep and superficial SSI will continue to increase as the number of fragility fractures increases, and therefore it is important that interventions optimise outcomes after surgery are evaluated. Pragmatic RCTs represent the highest level of evidence to assess the effectiveness of an orthopaedic intervention. However, there is a scarcity of well-designed and sufficiently powered RCTs of orthopaedic interventions, in particular with orthopaedic infections. Many orthopaedic trials fail to be delivered due to lack of scientific rigor in study design and execution. FHIT is a pragmatic trial which cannot blind the surgeon, however an appropriate sample size has been calculated, outcome assessors are blinded, clear inclusion and exclusion criteria defined, and information on statistical analysis considered appropriately. The FHIT trial has been designed to demonstrate a clinically important difference for patients, surgeons and within the wider setting of the NHS.

The primary aim of the FHIT trial is to determine if the addition of high dose antibiotic impregnated cement to the standard regime at Northumbria NHS trust can significantly reduce SSI at 12-months after hip hemiarthroplasty. The results from this trial will inform evidence-based recommendations for patients with an intracapsular fractured neck of femur treated with a hemiarthroplasty. If high dose antibiotic impregnated cement is found to be an effective intervention, its implementation into clinical practice could reduce SSI after hemiarthroplasty, and therefore improve long-term outcomes for patients suffering from this fragility fracture.

## Abbreviations

SSI: Surgical site infection; RCT: Randomised controlled trial; HPA: Health protection agency; NOF: Neck of femur fracture; NHS: National health service; THR: Total hip replacement; BP: Bipolar prosthesis; NHFD: National hip fracture database; FHIT: Fractured hip infection trial; CDC: Centers for disease control and prevention; ONS: Office of national statistics; DVT: Deep vein thrombosis; PE: Pulmonary embolism; TIA: Transient ischemic attack; GIB: Gastrointestinal bleeds; UR: Urinary retention; UTI: Urinary tract infection; MI: Myocardial infarction; RTI: Pneumonia; ARF: Acute renal failure; MCAR: Missing completely at random (MCAR); ITT: Intention-to-treat; DMC: Data monitoring committee.

## Competing interests

The authors declare that they have no competing interests.

## Authors’ contributions

All authors conceived and designed the study. All authors drafted the manuscript, revised it critically for important intellectual content and have given final approval of the version to be published. All authors read and approved the final manuscript.

## Pre-publication history

The pre-publication history for this paper can be accessed here:

http://www.biomedcentral.com/1471-2474/14/356/prepub
